# Philosophy of Nutrition, with Its Legal Aspect

**Published:** 1891-07

**Authors:** 


### Editorial, Etc.

PHILOSOPHY OF NUTRITION, WITH ITS LEGAL ASPECT.—At a recent meeting of the Akron, Ohio, Dental Society, Dr. Samuel D. Stewart read a very interesting and instructive paper on "The Philosophy of Nutrition with its Legal Aspect." He first spoke of the non-education and misuse of the sensory organs by man compared with the lower animals. An animal judges of the fitness of food by first smelling of it, and rejects it if not satisfied with it, while man takes it into his mouth first and often disposes of it so quickly that he hardly even tastes it. The principal part of Dr. Stewart's paper was to present some facts concerning the wholesale adulteration of foods and drugs, more especially foods, that enter into our every day diet and to call attention to the little protection afforded the people of this State compared with what is given in other states. It is a fact that Ohio is becoming the dumping-ground and sink-hole of manufacturers from all over the country for their foods and products which are unsaleable elsewhere. The Food Commission of Ohio is doing all that can be expected under the conditions that control it and much credit is due the chief and his able assistants. They were allowed by Legislature only $6,000 during the last year. Out of this the chief received $2,000 for salary and expenses and the remainder had to pay for assistant chemists and the many other expenses incidental to this kind of work. It is a wonder that so much was done with this mean, niggardly allowance. Massachusetts spent $10,-356 in one year, this being about $1.89 per test and their work has done much to increase the public health.

Minnesota spent $33,693.19 in the past year. The comparison speaks for itself.

The last report of the Ohio Commission shows good work among which may be noted the examination of canned goods which are being used more every day. They examined 19 cans of vegetables, eight cans of fruits, two of fish and one of milk. With the exception of the can of milk the contents of each can was found contaminated with the salts of tin in such quantities that a continued use of the contents would produce harmful results. These samples were not picked out, but were bought in open market of regular and reliable dealers and were standard brands.

Another important point spoken of was the use of preservations. While the use of them by a manufacturer is not such a fraud as adulteration the results are just as harmful. These are used in fruits, meats, fish, etc.

Baking powder also claimed the doctor's attention. Of the large number on the market there are but few which are pure and wholesome. Many contain alum in harmful quantities. Vinegar is adulterated so as to be harmful; so is jelly and nearly all if not all foods used in our every day life. Water, an important item in human welfare, while not adulterated intentionally is very often contaminated by the imperfect sewerage and some samples of waters which have been analyzed have been found to be simple sewerage that has found its way into wells after passing through a few feet of sand.

Massachusetts will spend $27,000 to test waters in her cities and towns and to test methods of disposing of her sewerage.

The question of water supply is of especial interest at the present time to the people of Akron. Our city water supply should be tested and analyzed oftener to protect the city. While quantity and constancy are desirable, purity is all important as water is often the cause of sickness and frequently of epidemics which almost devastate portions of a city.

It remains with the people to demand that these important subjects be remedied as they can be and it rests with those in public office to do their duty while the man who takes another's life is criminal, the man or corporation who, for the sake of the almighty dollar, causes the sale of impure goods, poisonous goods, which causes sickness and often make man a lifelong sufferer, is as great a one.

The doctor held the closest attention of the society for two hours and a condensed report cannot do him justice or begin to give an idea of the amount of facts he presented. The society heartily concurred with the speaker that this gross injustice needs attention, and that the people need to be agitated to realize the fact that they are daily taking into their systems that which poisons, causes sickness and often kills, and that it is time to demand of our Legislature bodies protection and security in our physical and moral rights.

